# Individualized Comprehensive Lifestyle Intervention in Patients Undergoing Chemotherapy with Curative or Palliative Intent: Who Participates?

**DOI:** 10.1371/journal.pone.0131355

**Published:** 2015-07-15

**Authors:** Karianne Vassbakk-Brovold, Sveinung Berntsen, Liv Fegran, Henrik Lian, Odd Mjåland, Svein Mjåland, Stephen Seiler, Christian Kersten

**Affiliations:** 1 Department of Oncology, Southern Hospital Trust, Kristiansand, Norway; 2 Department of Public Health, Sport and Nutrition, Faculty of Health Sport Sciences, University of Agder, Kristiansand, Norway; 3 Department of Health and Nursing Sciences, Faculty of Health and Sport Sciences, University of Agder, Kristiansand, Norway; 4 Department of Physical Medicine and Prevention, Southern Hospital Trust, Kristiansand, Norway; 5 Department of Surgery, Southern Hospital Trust, Kristiansand, Norway; Geisel School of Medicine at Dartmouth College, UNITED STATES

## Abstract

**Objective:**

Knowledge about determinants of participation in lifestyle interventions in cancer patients undergoing chemotherapy, particularly with palliative intent, remains poor. The objective of the present study was to identify determinants of participating in a 12 month individualized, comprehensive lifestyle intervention, focusing on diet, physical activity, mental stress and smoking cessation, in cancer patients receiving chemotherapy with curative or palliative intent. The secondary objective was to identify participation determinants 4 months into the study.

**Methods:**

Newly diagnosed cancer patients starting chemotherapy at the cancer center in Kristiansand/Norway (during a 16 month inclusion period) were screened. Demographic and medical data (age, sex, body mass index, education level, marital status, smoking status, Eastern Cooperative Oncology Group performance status (ECOG), diagnosis, tumor stage and treatment intention) was analyzed for screened patients.

**Results:**

100 of 161 invited patients participated. There were more females (69 vs. 48%; *P* = 0.004), breast cancer patients (46 vs. 25%; *P* = 0.007), non-smokers (87 vs. 74%; *P* = 0.041), younger (mean age 60 vs. 67 yrs; *P* < 0.001) and fitter (82 vs. 64% with EGOC 0; *P* = 0.036) participants vs. non-participants included. In multivariate logistic regression analyses, age (Odds Ratio 0.94, 95% Confidence Interval 0.91, 0.97) and smoking (0.42, 0.18, 0.99) were negatively associated with participation. After 4 months, 63 participants were still participating. Cancer type, smoking and age increased the probability of dropping out. Multivariate logistic regression revealed that age was the only significant determinant of 4 month participation (0.95, 0.91, 0.99). Patients aged >70 years were less likely to participate at baseline and 4 months.

**Conclusion:**

Individualized lifestyle interventions in cancer patients undergoing chemotherapy appear to facilitate a high participation rate that declines with increasing age; both during the enrollment process and completing the intervention. Neither oncologic nor socioeconomic variables deterred participation.

## Background

The age distribution of the Western population is right shifting, and as a consequence, both the incidence and prevalence of cancer are continuously rising [1]. Partly due to these demographic changes and in spite of improvements in diagnosis and treatment, cancer is one of the leading causes of death in economically developed countries [2]. Previous studies have demonstrated that newly diagnosed cancer patients seek a more active role in their course of treatment to regain empowerment during their illness [3–6]. This is consistent with our own observations from 2011, where 26 out of 27 consecutive oncology outpatients would have said yes to guidance focusing on their diet, physical activity and mental stress while undergoing chemotherapy if it existed (unpublished results). In parallel, it is increasingly recognized that the variation in clinical outcomes among cancer patients is not solely determined by the characteristics of intrinsic determinants of the cancer cells, but also by host factors such as metabolism and tumor inflammation [7,8]. Preclinical and a few clinical studies indicate that detrimental tumor metabolism and inflammation may be targeted via different lifestyle measures [9]. These measures specifically include changes in the patient`s diet [10], physical activity level [11], mental stress [12] and cigarette smoking [13]. Current nutrition and physical activity guidelines for cancer patients [14] have been based on interventions with focus on only one of these lifestyle measures, occasionally two [15], mainly after completion of their treatment with curative intent [16]. Further, documentation supporting comprehensive lifestyle interventions in cancer patients during chemotherapy, both with curative, but especially with palliative intent, remains poor [15,17]. Most importantly, the majority of lifestyle interventions to date have been conducted in a selected population of the healthiest, fittest, most educated patients undergoing curative chemotherapy, with inclusion of only a minority of the target population [16,18–23]. This selection bias may be due to a commonly applied *one size fits all*-approach, which does not consider the patients’ preferences, abilities or perceived barriers to behavior change [14,24]. Few studies have tried to adapt lifestyle interventions according to the participants’ own wishes and barriers. A description of patients that choose not to participate under such circumstances would be important, since cancer patients with poor lifestyle may garner substantial health benefits from lifestyle interventions [7–13]. Identification of participation determinants should include a description of the patients, as well as tumor and treatment characteristics [16].

The objective of the present study was to identify patient, tumor and treatment related determinants of participating in a 12 month individualized, comprehensive lifestyle intervention (I CAN), focusing on diet, physical activity, mental stress and cessation of cigarette smoking, in cancer patients receiving chemotherapy with either curative or palliative intent. The secondary objective was to identify determinants of participating 4 months into the study.

## Materials and Methods

### The I CAN study

The present study is part of I CAN, a feasibility study with the aim to increase population based adherence to healthy lifestyle behaviors of cancer patients undergoing chemotherapy.

I CAN offered individualized, comprehensive lifestyle interventions to patients starting chemotherapy with curative or palliative intent for all types of cancer at one oncology center in Kristiansand/Norway during January 2013-May 2014. The intervention focused on optimization of diet [25], physical activity level [14], stress management [26], and smoking cessation [27]. All invited patients were informed that the study was individualized so it was entirely up to them whether and to what extent they wished to adhere to the lifestyle advice they were given. The lifestyle supervisors tried to facilitate for the participants to achieve their personal goals for a healthier lifestyle. When included, participants received an information binder with numerous recipes for healthy food and drinks, physical activity suggestions, and tips on how to manage stress. The binder also included a list of symptoms and side effects associated with having cancer and tips on how to deal with these. Then, participants and relatives were invited to a voluntary grouped start-up course at baseline where participants and, if feasible, relatives, were educated in evidence based lifestyle recommendations and how to adopt a healthier lifestyle. This was followed by monthly individual sessions with a lifestyle supervisor over a 12-month period while the participants had appointments at the cancer centre to avoid that time and travel distance became participant barriers. During the sessions, the participant’s lifestyle habits (diet, physical activity, sedentary time, subjective stress, stress management, smoking habits, and alternative treatments) were assessed using questionnaires and responses were entered into electronic questionnaires by the lifestyle supervisors, using dedicated software developed to monitor self-report outcomes in participants after specific adaption for the I CAN study (GoTreatIT Cancer V1.0) [28]. Based on the score of the different questionnaires, GoTreatIT Cancer V1.0 automatically displayed the patients’ lifestyle scores. Further, GoTreatIT Cancer V1.0 provided feedback to each participant and regarding what was necessary to reach the participants’ personal goals, and the supervisor made practical suggestions as to how to increase adherence according to the participants’ individual preferences, barriers and abilities. Thus, the electronic reporting system both captured scientific data and facilitated the lifestyle supervising process.

The lifestyle supervisors were 2 oncology nurses employed at the cancer center and the first author. All were trained in the use of the electronic database GoTreatIT Cancer V1.0 [28] and the lifestyle recommendations for this feasibility study. Physical examination (body mass and waist circumference) and exploratory blood biomarker sampling were conducted bimonthly together with measures of each participant’s QOL on 4 occasions (baseline, 2 and 4 months, and end of the study).

### Participants and procedures

The Center for Cancer Treatment in Kristiansand provides chemotherapy for all cancer patients within a geographic area containing ca. 180.000 inhabitants. Patients were consecutively included during 16 months in a population based setting. Physicians and nurses at the outpatient clinic have daily meetings to discuss all the patients receiving chemotherapy the coming day. The first author was present at these meetings during the inclusion period to identify possible eligible study participants. Patients were screened on the following inclusion criteria: 1) age ≥ 18 years; 2) life expectancy ≥ 6 months; 3) Eastern Cooperative Oncology Group performance status (ECOG) ≤ 2; and 4) able to speak and read Norwegian. Eligible patients were contacted by one of the lifestyle supervisors on their second cycle of chemotherapy.

### Assessments of determinants

Demographic and medical information was collected via self-report and medical records. Medical data included date of birth, height (collected by the physicians before start-up of chemotherapy), tumor type (later categorized into 1 = breast cancer; 2 = colorectal cancer; 3 = prostate cancer; 4 = other cancer types–note: lung and gynecologic cancer patients do not receive chemotherapy at the study center), tumor stage (I-IV), ECOG (0–2, assessed by the patients’ contact physician) and treatment intention (curative or palliative). Self-reported data included marital status (0 = single, divorced or widowed; 1 = married or living together), cigarette smoking status (0 = nonsmoker; 1 = smoker) and education level (0 = high school or less; 1 = college/university) for the I CAN participants. Body mass (Mechanical scale, Seca 761, Birmingham, United Kingdom) was measured by the lifestyle supervisors for participants and obtained via medical records for non-participants. Body mass index (BMI) was calculated and BMI from 25 to 29.9 kg/m^2^ was considered overweight and BMI ≥ 30 kg/m^2^ obese [29]. All data from non-participants was obtained from their medical records.

### Ethical considerations

The study was conducted according to the guidelines of the Helsinki Declaration. The Regional Committee for Medical and Health Research Ethics, South-East approved the study (ref.no. 2012/1717/REK). Written informed consent was obtained from all patients before inclusion.

### Statistical methods

Descriptive characteristics are presented as mean and standard deviation (SD). Differences in means and proportions are analyzed by independent t-tests and Chi-square tests, respectively. Age, gender, marital status, education level, BMI, cigarette smoking, ECOG, treatment intention, tumor stage and tumor type were included in univariate analyses. Finally, logistic regression was performed to calculate Odds Ratios (OR) for participating for different medical and demographic characteristics and are presented as OR with 95% Confidence Intervals (CI). Due to multi-colinearity, gender, diagnosis type, tumor stage and treatment intention were not included in the same model. Variables associated with participation were included in the multivariate logistic regression analyses if p < 0.2. Dependent variables were then removed in a step-down fashion as suggested by Hosmer and Lemeshow [30], until statistical significance (p-value ≤ 0.05) was reached for the remaining determinants. Statistical analysis was performed with SPSS statistical software version 21 (SPSS Inc., Chicago, IL, USA).

## Results

### Recruitment

**Fig 1:**
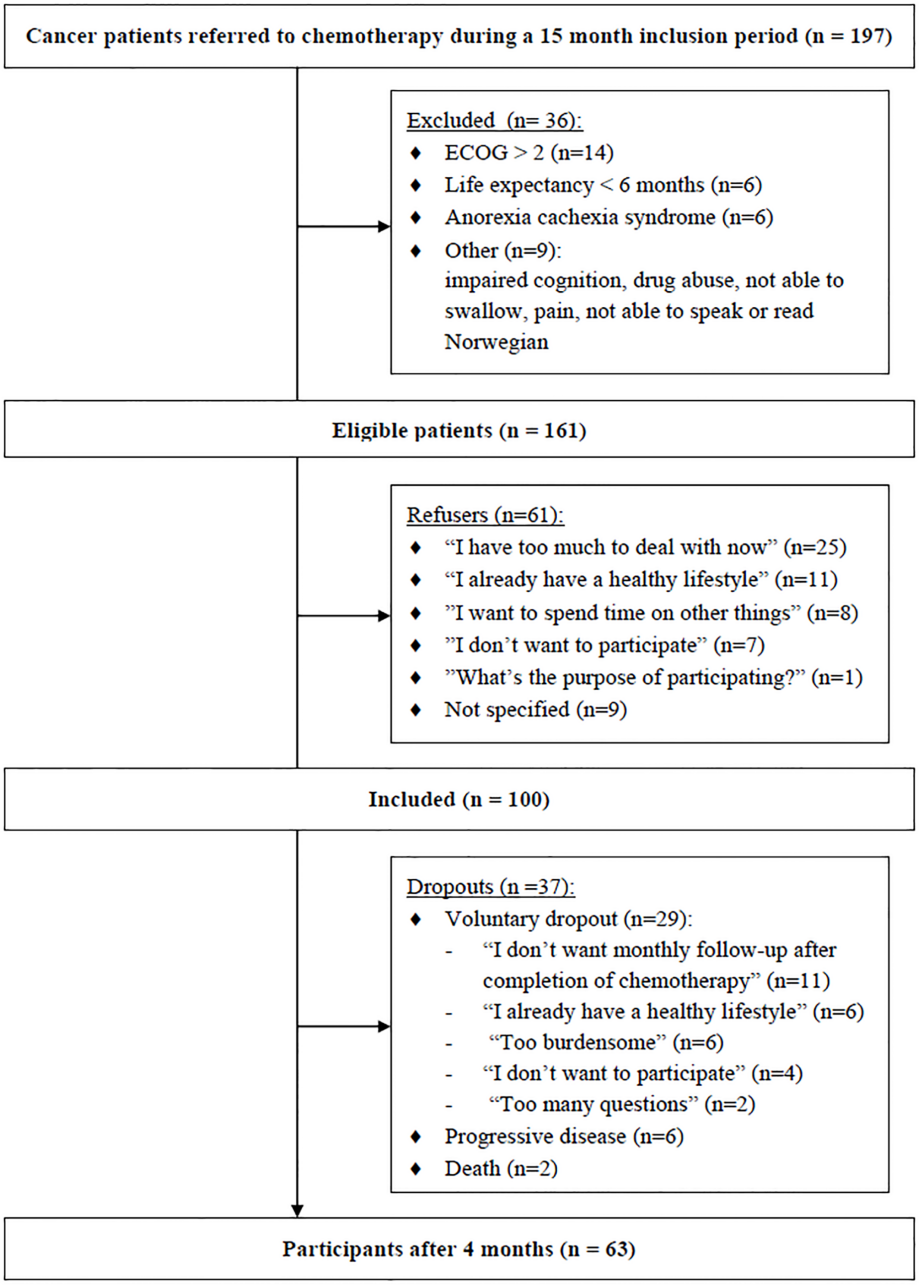


Recruitment is depicted in Fig 1. In total, 197 curative and palliative cancer patients starting chemotherapy were screened for participation in the I CAN study, of which 36 were excluded. Of the eligible 161 patients, 61 patients refused to participate. 52 of the 61 non-participants provided a reason for their refusal (S1 Fig). After exclusions and refusals, 100 of 161 eligible patients, with 14 different tumor types, were recruited and completed the baseline measurements.

### Determinants of participation in the I CAN study

There were significant differences between I CAN participants and the 61 eligible non-participants for age, gender, cigarette smoking, ECOG, and diagnosis (S1 Table).

In multivariate logistic regression analyses, age and cigarette smoking were associated with participation in the I CAN study (Nagelkerke 0.16) (S2 Table). Smokers were 58% less likely to participate and post hoc-analysis revealed a dose-response relationship for age; patients aged ≥ 70 years were 80% less likely to participate (0.20 (0.09, 0.48)) compared to patients < 59 years (S2 Fig). Gender, oncologic (ECOG, tumor stage, tumor type or treatment intention), and socioeconomic variables were not associated with participation.

### Determinants of participating after 4 months

After 4 months, 37 participants had dropped out voluntarily (n = 29), due to death (n = 2) or progressive disease (n = 6) (S1 Fig). Age, cigarette smoking and type of diagnosis increased the probability of dropping out (S3 Table).

In multivariate logistic regression analyses, younger age was associated with participation in the I CAN-study after 4 months (Nagelkerke 0.10) (S4 Table). Age increased the probability of dropout by 5% for every year the participant aged or 71% for patients >70 years compared to patients < 59 years (0.29 (0.09, 0.89)). Participation after 4 months was not associated with gender, oncologic (ECOG, tumor stage, tumor type or treatment intention), socioeconomic variables or smoking status.

## Discussion

In the present population based Norwegian study, > 60% of newly diagnosed cancer patients with a life expectancy of more than six months agreed to participate in an individualized comprehensive lifestyle intervention while undergoing chemotherapy with either curative or palliative intent. Four months into the study, 63% of the included 100 participants were still participating. Increasing age and cigarette smoking deterred participation in the I CAN study. Age was the only statistically significant dropout determinant 4 months into the study. Importantly, neither gender, oncologic variables (tumor type, treatment intention, tumor stage, ECOG) nor socioeconomic status were associated with participation at baseline or after 4 months.

One important determinant of both refusing to participate in the I CAN study and 4 month dropout was age, particularly being older than 70 years. Contrary to our 4 month findings, Courneya and co-workers [19] revealed no associations between age and exercise adherence in their 17 week exercise intervention in breast cancer patients with a mean age of 49 years (range: 25–78 years). The results of Oldervoll et al. [31], however, support our findings with significantly lower age reported among participants vs. non-participants in their exercise intervention among patients undergoing palliative chemotherapy. This is important findings, since cancer primarily is a disease of the elderly [1]. In Norway, three out of four new cancer cases are both in men and women over the age of 60 years [32] and half are diagnosed at ages 70 or older [33]. Previous research has pointed out the many barriers of older adults for behavior change [34,35]. In the study of Sallis et al. [34], some of the most common reasons for not adopting a more physically active lifestyle in healthy older adults included poor health, low self-efficacy, perceived lack of time, lack of self-motivation and lack of self-management skills such as the ability to set personal goals or monitor their own progress. The I CAN-study tried to adjust for the underrepresentation of senior cancer patients in lifestyle interventions by informing the eligible patients about potential health benefits by changing their lifestyles and how individualized recommendations would be given. The patients were also informed that it was entirely up to them how much time and effort they wanted to devote to this project and as a timesaving feature, assessments of their behaviors would be conducted monthly while receiving chemotherapy at the cancer centre. Additionally, the GoTreatIT was adapted specifically for the present study to ease the question burden on the participants and to possibly accommodate the senior population; in dialogue with the participants the lifestyle supervisor simultaneously completed the questionnaires instead of making the participants fill in all the forms by hand. Monitoring of own progress was greatly facilitated by this software. The mean age of the participants in the present study was 60 years at baseline and 57 years 4 months into the study; somewhat older compared to the patients in previous lifestyle interventions in cancer patients [16,19,20,23,36,37]. A reason for this relatively young age in previous lifestyle interventions may be that most lifestyle interventions are conducted in breast cancer patients, which are a younger population compared to other cancer populations, i.e. prostate cancer patients and colorectal cancer patients [33]. Breast cancer patients made up 46% of the I CAN participants. Importantly, this may be one reason why participants in both the present and previous lifestyle interventions often are younger than the average cancer patient [38].

Cigarette smoking was the second determinant of participation in the I CAN study but was no longer significantly associated with 4 month participation. Smokers refused to participate in the I CAN study more often than the non-smokers. Our data is consistent with the findings of Courneya et al. [19], who reported higher exercise adherence among non-smokers. Only 13% of the participants in the present study were smoking at the time of the baseline measurements, which is less compared to the adult Norwegian population where 26% are currently smoking, either on a daily or occasional basis [39]. Among the non-participants, 26% were current smokers. The proportion of smokers reported in the present study is in line with the findings in the multi-modal exercise intervention of Adamsen et al. [16], who reported that 14% of the patients were current smokers. In the exercise intervention of Midtgaard et al. [36], 23% of the participants were current smokers. After 4 months in the present study, 8 of the smokers had dropped out voluntarily (n = 5), due to death (n = 1) or progressive disease (n = 2). The remaining 5 smokers were still smoking after 4 months and did not want to quit smoking. Importantly, patients were not required to quit smoking to participate in the present study; focus on improving other aspects of the participants’ lifestyle was rather prioritized. Surprisingly, few lifestyle interventions in cancer patients have reported participant smoking status.

In 2011, all but one of the asked patients at our outpatient clinic wished to receive guidance regarding their diet, physical activity and mental stress while undergoing chemotherapy (unpublished results). When I CAN finally was offered to the patients, 38% of the patients did not wish to participate after all. Still, an inclusion of 62% of eligible patients in the present study is higher than reported in other lifestyle interventions in cancer patients during their treatment [18–22], despite that the I CAN-study included patients currently undergoing chemotherapy with either curative or palliative intent. The reason might be due to an individualization of the present intervention study, where a *one size fits all*-approach commonly have been applied in previous lifestyle interventions in cancer patients. Such a standard prescription approach is characterized by rigid interventions in which all participants are instructed to follow the same prescription, at the same place and at the same time [22,24,37]. The participants in the exercise intervention of Courneya et al. [38], reported that disease and treatment-related barriers (feeling sick, fatigue, loss of interest, vacation, nausea/vomiting, work issues and pain) accounted for more than 50% of the missed exercise sessions. This illustrates the need for strategies to overcome these barriers. Individualization may be one strategy. Buffart and colleagues [24] confirmed this by suggesting moving from a *one size fits all*-approach to more specific guidelines tailored to the individual patients’ characteristics, needs, capabilities and preferences. In the present study, participants decided which of the individualized lifestyle recommendations they were willing or able to adhere to, and to what extent, with recommendations adjusted accordingly. Additionally, participants were informed that the study appointments would take place on the same day as their chemotherapy treatment at the cancer center; the study was thus facilitated to be time-effective for the participants. Still, the most common barrier for not participating in the I CAN-study, was “having too much to deal with”. Importantly, barriers to adopting new behaviors may differ from the barriers to adhering to current behaviors [19].

Focusing on curative patients, 68% agreed to participate in our study. This is almost three times the inclusion rate compared to the study of Ornish et al. [22], which to our knowledge is the only larger comprehensive lifestyle intervention in cancer patients published to date, where only 24% of the eligible low-risk prostate cancer patients were included. It is noteworthy that the participants in the study of Ornish and colleagues were not undergoing chemotherapy, but active surveillance for their cancers. The determinants of lifestyle change during chemotherapy may be quite different than during active surveillance or during survivorship, because of the known toxicities of these treatments [20]. The large difference in inclusion rate between the present lifestyle intervention and the intervention of Ornish et al. may again be explained by our individualized approach. In line with this argument, three of four eligible patients in the study of Ornish and colleagues declined to participate after learning more about the study.

Very few lifestyle intervention studies have been conducted in palliative patients. Thus, the inclusion of metastatic cancer patients undergoing chemotherapy with palliative intent is an important strength of the present study. Our inclusion rate of 55% among those receiving palliative treatment is in line with Oldervoll et al. [31], who included 63% of the eligible palliative patients to their 6-week exercise intervention. However and importantly, only 14% of the patients included to their exercise intervention were undergoing chemotherapy at the time of their trial (personal communication), compared to 100% of our patients.

It is important to note that oncologic or socioeconomic variables in the present study did not influence participation. Determinants that determined participation in univariate analyses, but became non-significant in multivariate analyses included gender, ECOG and diagnosis. Variables that did not determine participation, even in univariate analyses, included marital status, education level, BMI, tumor stage and treatment intention (palliative vs. curative). The latter is especially noteworthy, since this population often asks for empowerment tools within the lifestyle spectrum and may experience some benefits from improving their lifestyle, but is rarely targeted in studies [40].

The study of Courneya et al. [20], supports our findings with regard to socioeconomic status; neither education nor marital status was associated with exercise adherence among breast cancer patients undergoing chemotherapy. In contrast, another study of Courneya and colleagues [19] revealed univariate associations between education and exercise adherence in breast cancer patients undergoing chemotherapy. In other words, it remains unclear whether socioeconomic variables affect lifestyle behavior among cancer patients undergoing chemotherapy and if the results differ when we are measuring determinants of participation versus adherence.

One important strength of the present study include being the first population-based study to prospectively examine the determinants of participating in an individualized, comprehensive lifestyle intervention in cancer patients receiving both curative and palliative chemotherapy. Therefore, determinants of participation could, for the first time, be compared directly between these two groups of patients. Secondly, the present study also collected demographic, medical and physiologic data on the patients who declined to participate, giving us the opportunity to describe a previously understudied patient group. Another strength of the present study was the close collaboration between the oncologic professionals and the lifestyle intervention researchers. The head of both the oncologic and surgical departments were two of the initiators of the study, which signalized the importance of this study to the rest of the clinical units. The close collaboration also simplified and quality assured the inclusion process by giving the first author access to all relevant patients, consequently avoiding the “gatekeeping” issue where the health care professionals try to protect the patients from potentially unnecessary and exhausting strains [31].

One limitation of the present study is that the participants’ attitudes toward participation were not reported; neither were the non-participants’ attitude against participation other than their verbal reasons for not participating. Additionally, assessments of the non-participants’ lifestyle habits at baseline would be of great interest to identify possible lifestyle related determinants for participating. The identified participant determinants at baseline and 4 months explained a minority of the variance of willingness to participate, suggesting that other important factors, such as motivational and lifestyle determinants, most likely affect participation. Some likely factors include motivational factors, suggested by Courneya and colleagues [19], which we did not collect from I CAN-participants or non-participants. Another limitation of the present study is the low number of smokers included, which increases the possibilities of type 2 errors. At 4 months smoking was no longer significantly associated with participation. However, with a larger sample size smoking status would most likely have been a significant determinant of 4 month participation.

Before the start-up of the I CAN-study, 27 patients starting conventional treatment at the cancer center, including elderly patients, were asked if they would be willing to participate in a lifestyle intervention if it existed. In that pilot study, 26 of 27 patients said yes (own unpublished results). Our results from the present study show that older patients and smokers refused to participate more often than younger patients and non-smokers. After 4 months, age was the only significant identified participant determinant. In other words; the patients who might experience the largest benefits of improving their lifestyles [7–13] refused to participate in the present study. Despite statistically non-significant, a significant proportion of the smokers withdrew from the present study. Nevertheless, 5 of the 13 included smokers included at baseline were still participating after 4 months; all still smoking. It is thus important to provide patients with accurate information when informing about the study and what is required of them and not. This was provided in the present study, but one can question how much of this information that actually reaches the patients who have just been diagnosed with cancer, starting up chemotherapy and who are loaded with information from both nurses and doctors. Thus, it remains challenging to adapt future lifestyle interventions in order to include the patients that do not wish to participate. This is an important task, since these patients may gain most health benefits from lifestyle changes. When the I CAN study was developed, the goal was to design an individualized intervention that included also the less healthy part of the population by offering a low-threshold intervention. Despite of a comparably high participation rate in both early stage and metastatic cancer patients, the I CAN study still fails to include the elderly and less healthy patients compared to the younger non-smokers. This raises the question of how we could design individually adapted interventions for this part of the population. However, it appears noteworthy that in terms of accommodating the individual patients’ needs, it might be neither possible nor desirable from the patients’ perspective to achieve a 100% inclusion rate. Nonetheless and importantly, researchers should still be aware of existing barriers in planning and designing future lifestyle interventions to achieve the highest possible inclusion in this less studied part of the population who might benefit the most from altering their lifestyles.

## Conclusions

Individualized lifestyle interventions in cancer patients undergoing chemotherapy appear to facilitate a high participation rate that declines with increasing age; both during the enrollment process and while completing the intervention. Neither oncologic nor socioeconomic variables deterred participation. Importantly, cancer patients undergoing chemotherapy with palliative intent are willing to participate in lifestyle interventions studies. Thus, studies with overall survival as an endpoint may be feasible and targeting patients receiving palliative chemotherapy with a reasonable life expectancy is warranted.

## Supporting Information

S1 Fig Flow chart of the patient recruitment and participation after 4 months. (TIF)

S2 Fig Odds Ratios (95% CI) for participating in I CAN by age (in categories) and cigarette smoking. (TIF)

S1 File The SPSS data file containing the I CAN participants’ and non-participants’ anonymous medical and demographic information. (SAV)

S1 Table (DOCX)

S2 Table (DOCX)

S3 Table (DOCX)

S4 Table (DOCX)
