# Supplementary figures and images for: Individualized Comprehensive Lifestyle Intervention in Patients Undergoing Chemotherapy with Curative or Palliative Intent: Who Participates?
Source: PLoS One. 2015 Jul 15;10(7):e0131355. doi: 10.1371/journal.pone.0131355 (PMC4503483; doi:10.1371/journal.pone.0131355)

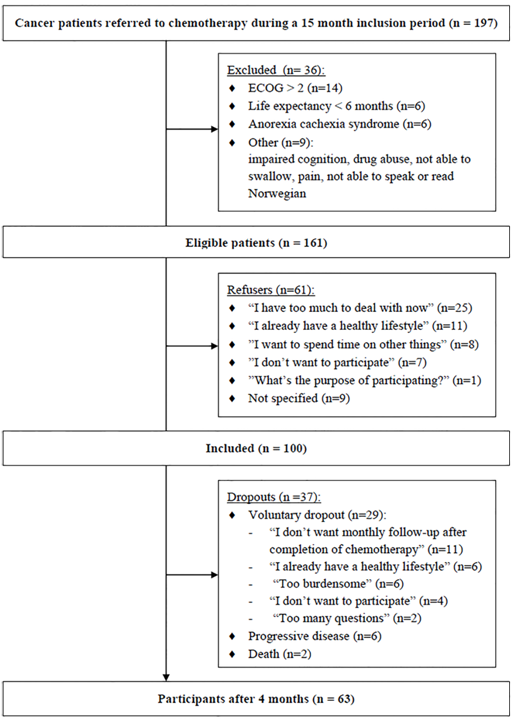

Supplement: S1 Fig — (TIF) [file pone.0131355.s001.tif]

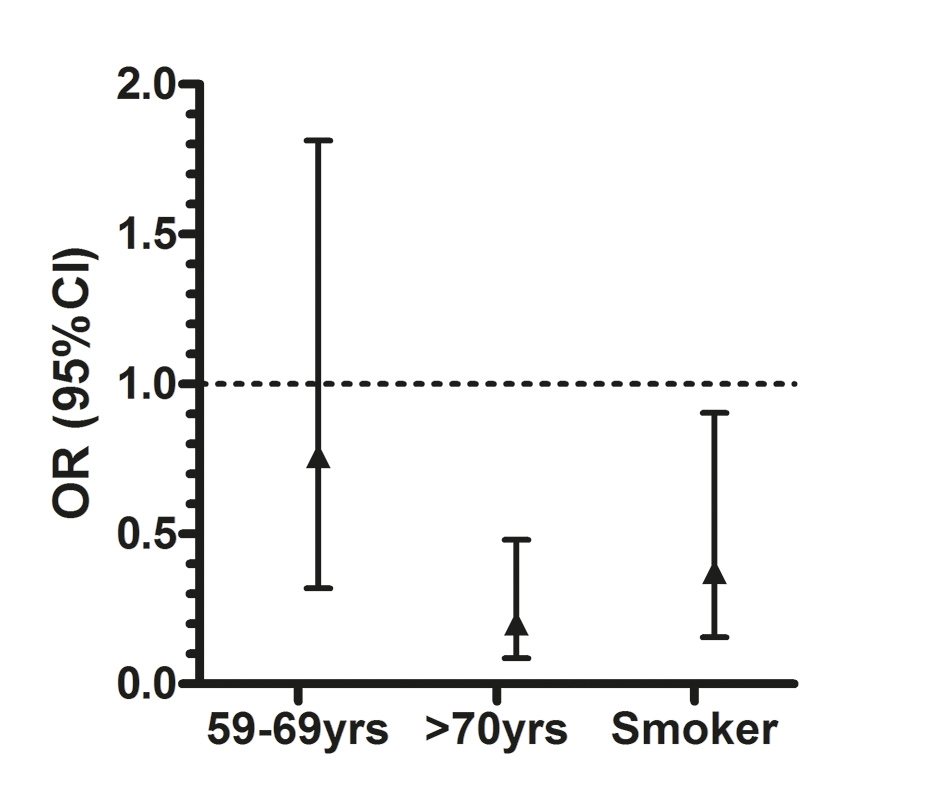

Supplement: S2 Fig — (TIF) [file pone.0131355.s002.tif]
