# Supplementary material for: Individualized Comprehensive Lifestyle Intervention in Patients Undergoing Chemotherapy with Curative or Palliative Intent: Who Participates?
Source: PLoS One. 2015 Jul 15;10(7):e0131355. doi: 10.1371/journal.pone.0131355 (PMC4503483; doi:10.1371/journal.pone.0131355)
Supplement: S1 Table — (DOCX) [file pone.0131355.s004.docx]

**S1 Table.** Participants’ demographic and medical characteristics (frequencies and percentages in parenthesis unless otherwise stated*).

|  | I CAN participants | | Non-participants | |  |
| --- | --- | --- | --- | --- | --- |
|  | **n=100** | **(%)** | **n=61** | **(%)** | ***P-value*** |
| Age, mean (SD)* | 60 (12) | | 67 (11) | | <.001 |
| Gender  Men  Women | 31  69 | (31)  (69) | 32  29 | (52)  (48) | .004 |
| Marital status  Married/living together  Single/divorced/widowed | 80  20 | (80)  (20) | **  48  12 | (80)  (20) | 1.000 |
| Education level  High school or less  College/university | **  47  50 | (48)  (52) | **  27  28 | (49)  (51) | .940 |
| BMI  <18.5  18.5-24.9  25-39.9  >30 | 3  45  40  12 | (3)  (45)  (40)  (12) | **  0  37  16  6 | (0)  (63)  (27)  (10) | .117 |
| Cigarette smoking  Smoker  Nonsmoker | 13  87 | (13)  (87) | **  15  43 | (26)  (74) | .041 |
| ECOG  0  1  2 | 82  16  2 | (82)  (16)  (2) | **  37  18  3 | (64)  (31)  (5) | .036 |
| Treatment intention  Curative  Palliative | 60  40 | (60)  (40) | 28  33 | (46)  (54) | .081 |
| Tumor stage  I  II  III  IV | 16  20  23  41 | (16)  (20)  (23)  (41) | **  6  4  17  33 | (10)  (7)  (28)  (55) | .059 |
| Diagnosis  Breast cancer  Colorectal cancer  Prostate cancer  Other | 46  31  4  19 | (46)  (31)  (4)  (19) | 15  20  10  16 | (25)  (33)  (16)  (26) | .007 |

**Missing data
