# Supplementary material for: Individualized Comprehensive Lifestyle Intervention in Patients Undergoing Chemotherapy with Curative or Palliative Intent: Who Participates?
Source: PLoS One. 2015 Jul 15;10(7):e0131355. doi: 10.1371/journal.pone.0131355 (PMC4503483; doi:10.1371/journal.pone.0131355)
Supplement: S2 Table — (DOCX) [file pone.0131355.s005.docx]

**S2 Table.** Regression summaries for univariate- and multivariate analyses with the dependent variable participation (OR and 95% CI).

|  | Bivariate analyses | Intermediate multivariate analyses | Final multivariate analyses |
| --- | --- | --- | --- |
| Age | 0.94 (0.91, 0.97) | 0.96 (0.92, 0.99) | 0.94 (0.91, 0.97) |
| Male | 0.39 (0.20, 0.75) | 0.55 (0.25, 1.18) | - |
| Single/divorced/widowed | 1.00 (0.45, 2.23) | - | - |
| High school or less | 0.98 (0.50, 1.89) | - | - |
| BMI | 1.06, (0.98, 1.15) | 1.03 (0.95, 1.13) | - |
| Smoking cigarettes | 0.43 (0.19, 0.98) | 0.49 (0.19, 1.25) | 0.42 (0.18, 0.99) |
| ECOG 1-2 | 0.39, (0.19, 0.81) | 0.63 (0.26, 1.49) | - |
| Palliative treatment | 0.57 (0.30, 1.08) | 1.03 (0.46, 2.32) | - |

* References: Women, Married/living together, high level of education, nonsmoking, ECOG = 0 and curative patients
