# Supplementary material for: Individualized Comprehensive Lifestyle Intervention in Patients Undergoing Chemotherapy with Curative or Palliative Intent: Who Participates?
Source: PLoS One. 2015 Jul 15;10(7):e0131355. doi: 10.1371/journal.pone.0131355 (PMC4503483; doi:10.1371/journal.pone.0131355)
Supplement: S3 Table — (DOCX) [file pone.0131355.s006.docx]

**S3 Table.** Demographic differences between participants and dropouts 4 months into the study (frequencies and percentages in parenthesis unless otherwise stated*).

|  | I CAN participants | | Dropouts | |  |
| --- | --- | --- | --- | --- | --- |
|  | **n=63** | **(%)** | **n=37** | **(%)** | ***P-value*** |
| Age, mean (SD)* | 57 (12) | | 64 (11) | | .006 |
| Gender  Men  Women | 17  46 | (27)  (73) | 13  24 | (35)  (65) | .390 |
| Marital status  Married/living together  Single/divorced/widowed | 52  11 | (83)  (17) | 28  9 | (76)  (24) | .407 |
| Education level  High school or less  College/university | **  26  35 | (43)  (57) | 21  15 | (58)  (42) | .135 |
| BMI  <18.5  18.5-24.9  25-39.9  >30 | 1  28  28  6 | (2)  (44)  (44)  (10) | 2  17  12  6 | (5)  (46)  (32)  (16) | .414 |
| Cigarette smoking  Smoker  Nonsmoker | 5  58 | (8)  (92) | 8  29 | (22)  (78) | .049 |
| ECOG  0  1  2 | 49  12  2 | (78)  (19)  (3) | 33  4  0 | (89)  (11)  (0) | .282 |
| Treatment intention  Curative  Palliative | 37  26 | (59)  (41) | 23  14 | (62)  (38) | .735 |
| Tumor stage  I  II  III  IV | 11  14  12  26 | (18)  (22)  (19)  (41) | 5  6  11  15 | (14)  (16)  (30)  (41) | .613 |
| Diagnosis  Breast cancer  Colorectal cancer  Prostate cancer  Other | 34  19  4  6 | (54)  (30)  (6)  (10) | 12  12  0  13 | (32)  (32)  (0)  (35) | .005 |

** Missing data
