# Supplementary material for: Individualized Comprehensive Lifestyle Intervention in Patients Undergoing Chemotherapy with Curative or Palliative Intent: Who Participates?
Source: PLoS One. 2015 Jul 15;10(7):e0131355. doi: 10.1371/journal.pone.0131355 (PMC4503483; doi:10.1371/journal.pone.0131355)
Supplement: S4 Table — (DOCX) [file pone.0131355.s007.docx]

**S4 Table.** Regression summaries for univariate- and multivariate analyses with the dependent variable participation after 4 months (OR and 95% CI).

|  | Bivariate analyses | Intermediate multivariate analyses | Final multivariate analyses |
| --- | --- | --- | --- |
| Age | 0.95 (0.91, 0.99) | 0.93 (0.88, 0.97) | 0.95 (0.91, 0.99) |
| Male | 0.68 (0.29, 1.64) | - | - |
| Single/divorced/widowed | 0.66 (0.24, 1.78) | - | - |
| High school or less | 0.53 (0.23, 1.22) | 0.75 (0.28, 2.02) | - |
| BMI | 0.98 (0.98, 1.08) | - | - |
| Smoking cigarettes | 0.31 (0.94, 1.04) | 0.22 (0.05, 1.02) | - |
| ECOG 1-2 | 2.36 (0.71, 7.79) | 7.14 (1.69, 30.12) | - |
| Palliative treatment | 1.15 (0.50, 2.65) | - | - |

* References: High level of education, nonsmoking and ECOG = 0.
